# Editorial: Imaging of bone cancers – novel methodologies and optimizations

**DOI:** 10.3389/fonc.2023.1148663

**Published:** 2023-02-17

**Authors:** Carmelo Messina, Filippo Del Grande

**Affiliations:** ^1^ Unità Operativa di Radiologia Diagnostica e Interventistica, IRCCS Istituto Ortopedico Galeazzi, Milan, Italy; ^2^ Dipartimento di Scienze Biomediche per la Salute, Università degli Studi di Milano, Milan, Italy; ^3^ Istituto di Imaging della Svizzera Italiana (IIMSI), Ente Ospedaliero Cantonale (EOC), Lugano, Switzerland; ^4^ Facoltà di Scienze Biomediche, Università della Svizzera Italiana, Lugano, Switzerland

**Keywords:** bone tumours, bone metastasis, artificial intelligence, radiomics, machine learning, imaging, nuclear medicine

Bone pathology include a wide range of diseases, such as neoplastic disorders, which are uncommon but carry high risk of morbidity and mortality (1). Bone metastases are much more common compared to primary bone malignant tumours and may represent the first manifestation in oncologic patients.

Imaging is essential for correct diagnosis and for disease staging. Unfortunately, conventional imaging techniques in musculoskeletal tumour assessment may suffer of several limitations (2–4). Advanced imaging techniques, such as DWI, perfusions imaging and recent emerging applications such as radiomics and artificial intelligence (AI) play an important role for correct diagnosis.

We propose the topic “Imaging of Bone Cancers – Novel Methodologies and Optimizations”, to further expand the knowledge on recent developments of imaging in bone cancer. Eight papers were published, including two systematic reviews, five original articles, and one case presentation.

Wu et al. performed a systematic review and meta-analysis to evaluate the ability ^99m^Tc-MIBI, an imaging agent commonly used for myocardial perfusion, in assessing the preoperative response of osteosarcoma patients to neoadjuvant chemotherapy. Overall, 8 articles were included (189 osteosarcoma patients), in a study that represented the first meta-analysis on this topic. The uptake change ratio (ΔUR) of ^99m^Tc-MIBI (before and after chemotherapy) and the washout rate (WR) before chemotherapy were evaluated. Based on the values of AUC and pooled diagnostic odds ratio (DOR), both the ΔUR and WR from ^99m^Tc-MIBI were valuable in the preoperative assessment of the histological response of osteosarcoma patients to neoadjuvant chemotherapy, with ΔUR showing better diagnostic accuracy than WR.

Another systematic review was conducted by the group of Li et al. The paper focused on the analysis of [^68^Ga]Ga-DOTAFAPI-04 and [^18^F]FDG PET/CT for the diagnosis of bone metastases. Eight studies were included, for a total of 358 patients. Better diagnostic performance emerged for [^68^Ga]Ga-DOTA-FAPI-04, especially in terms of sensitivity, while the specificity [^18^F]FDG still remains higher mainly due to false positive results of [^68^Ga]Ga-DOTA-FAPI-04 for benign inflammation reactions. The results of this meta-analysis show how further studies are warranted to fully understand the comparative values of these two agents in detecting bone metastases.

Staying with nuclear medicine, Guo et al. published an original study for the assessing the potential

application of ^68^Ga-P15-041, a novel bone-seeking radiotracer, for clinical PET/CT imaging in the detection of bone cancer metastases. Authors also compared its efficacy to that of the most commonly used ^99m^Tc-MDP. The study was conducted on 51 patients, for a total of 174 bone metastatic sites. According to the study results, ^68^Ga-P15-041 PET/TC showed higher accuracy values compared to ^99m^Tc-MDP scintigraphy in detecting bone metastases, with higher values of accuracy. Therefore, PET/CT with ^68^Ga-P15-041 could become a valuable nuclear medicine procedure to detect bone metastases.

The paper by Du et al. also focused on bone osteosarcoma. In fact, they presented a case of a 13 years-old female patient suffering of a large pelvic osteosarcoma, in which they propose a novel artificial intelligence (AI)-assisted CT/MRI image fusion technique to build a personalized 3-D model for preoperative assessment of tumour margin. For the first time, this fusion model allowed for a more detailed anatomical study, by putting together the very good bony detail of CT images together with the accurate information on soft tissue mass by MRI. According to authors, this technique may also be applied in the future to other tumours, especially those occurring in small and irregular bones.

The Research Topic also included studies about more conventional images. The paper by Wang et al., compared ultrasound to CT, X-ray and ^99^mTc-MDP bone scan in the diagnosis of local recurrence after limb salvage in patients who underwent primary bone surgery. This retrospective study reviewed a total of 288 cases, showing better sensitivity and accuracy values for US compared to X-ray. Of note, also CT and ^99^mTc-MDP bone scan were superior to X-ray. Despite the study did not include data from PET/CT and MRI, and the fact that US is an operator-dependent technique, US showed to be a reliable technique for postoperative surveillance of primary bone tumours. Multicenter prospective studies are warranted in the future to further confirm the results of this study.

The collection also included an interesting research paper by Chen et al., which investigated a novel deep-learning-based method called Multi-view Attention-Guided Network (MAGN) for differentiating between spine metastases from multiple myeloma (MM) and lung cancer (LC). The MAGN model, built on three-plane contrast-enhanced T1WI (CET1) sequences on 217 patients, comparing its performance with other radiomics model and the visual radiologist assessment. Interestingly, the MAGN method outperformed both the radiomics method and radiologist assessment in differentiating MM and LC spine metastases. This very good performance is attributable to the peculiarity of MAGN method in extracting more comprehensive spinal disease features, representing a step forward in radiomic analysis for precision medicine.

Spinal metastasis was also the focus of the research paper by Zhang et al. with the purpose of investigating the value of intravoxel incoherent motion (IVIM) from MRI to discriminate spinal metastases from tuberculous spondylitis (TS). IVIM is a non-invasive MRI tool that visualize

microscopic motions of water providing information of molecular diffusion coefficients (ADC_slow_), perfusion-related diffusion (ADC_fast_), and the perfusion fraction (*f*). IVIM was applied in 50 patients with metastases and 20 with TS, showing significant differences only for the ADC_fast_ and *f* parameters. This study, despite the small sample size, can put the basis for further developments in the application of IVIM in the oncology field.

Finally, the research paper by Gitto et al. focused on MRI radiomics of skeletal

Ewing sarcoma (ES), with the aim of investigating feature reproducibility and machine learning prediction of response to neoadjuvant chemotherapy. The retrospective study was performed on 30 patients with histologically-proven skeletal ES and focused on feature reproducibility of 2D and 3D regions of interest. Compared to 2D approach, 3D MRI radiomics had superior reproducibility and higher accuracy in predicting response to neoadjuvant chemotherapy. This study highlighted that, despite 3D segmentations are much time-consuming than 2D, their use should be preferred.

## Author contributions

All authors listed have made a substantial, direct, and intellectual contribution to the work and approved it for publication.
